# Prevalence and antibiotic resistance of *Escherichia coli* O157:H7 in beef at a commercial slaughterhouse in Moro, Kwara State, Nigeria

**DOI:** 10.1099/acmi.0.000289

**Published:** 2021-11-11

**Authors:** Busayo I. Ajuwon, Sola K. Babatunde, Olatunji M. Kolawole, Adeyinka E. Ajiboye, Abosede H. Lawal

**Affiliations:** ^1^​ National Centre for Epidemiology and Population Health, Research School of Population Health, College of Health and Medicine, The Australian National University, Canberra, ACT 2601, Australia; ^2^​ Department of Biosciences and Biotechnology, Faculty of Pure and Applied Sciences, Kwara State University, Malete, Nigeria; ^3^​ Department of Natural and Environmental Sciences, Faculty of Science, Crown-Hill University, Ilorin, Kwara State, Nigeria; ^4^​ Department of Microbiology, Faculty of Life Sciences, University of Ilorin, Ilorin, Nigeria; ^5^​ Centre for Occupational and Environmental Public Health, Cardiff School of Sport and Health Sciences, Cardiff Metropolitan University, Cardiff, UK

**Keywords:** gastroenteritis, *E. coli *O157:H7, prevalence, beef, antibiotic susceptibility

## Abstract

**Background:**

Gastroenteritis due to foodborne disease is a leading cause of death in developing countries. In Nigeria, there is an increasing demand for beef. Yet, there is no surveillance for *Escherichia coli* O157:H7 contamination of raw beef and little is known about the carriage of this pathogen in Nigeria’s livestock.

**Methods:**

A total of 415 samples, including 180 cow carcass swabs, 180 caecal content samples, 16 water samples, 25 hand swabs and 14 knife swabs were collected at a large abattoir in the Moro region of Kwara State, Nigeria. The samples were enriched in modified tryptone broth containing novobiocine, and plated onto Sorbitol–MacConkey agar (Oxoid SR0172E) supplemented with 0.05 mg l^−1^ cefixime and 2.5 mg l^−1^ potassium tellurite (Oxoid) (CT-SMAC). Indole-producing isolates were confirmed serologically by serotyping with antisera specific for the O157 and H7 antigens. The *E. coli* O157:H7 isolates were further tested for their susceptibility to antibiotic agents using the disc diffusion method. Commercially available Gram-negative multi-discs (Oxoid) comprising nitrofurantoin (30 µg), ampicillin (5 µg), ceftazidime (30 µg), gentamicin (10 µg), ciprofloxacin (5 µg), augmentin (30 µg), ofloxacin (5 µg) and cefuroxime (30 µg) were tested.

**Results:**

Overall, 16 (3.9 %) samples were contaminated with *E. coli* O157:H7, of which 10 (5.6 %) were isolated from carcass swabs, 4 (2.2 %) from caecal content samples and 2 (12.5 %) from water. All isolates were multidrug-resistant (MDR), with resistance to ampicillin, ceftazidime and cefuroxime being the most common.

**Conclusion:**

This study provides evidence to suggest that *E. coli* O157:H7 exists in the beef production chain. The pathogen reveals a high frequency of multidrug resistance, suggesting that consumers and handlers of such meat are at risk of contracting antibiotic-resistant *E. coli* O157:H7-associated foodborne disease. Routine monitoring of antibiotic resistance is critical to uncovering novel therapeutic strategies that will help inform clinical practice guidelines.

## Background

The burden of foodborne diseases in Africa has become a major public health concern due to Africa’s numerous infrastructural challenges. Enterohaemorrhagic *Escherichia coli* O157:H7 is one of the most important foodborne pathogens. It often causes significant mortality among the human population [1]. Typical illnesses as a result of this pathogen can be life threatening. Susceptible individuals show a range of symptoms, including haemorrhagic colitis and other complications, such as haemolytic uremic syndrome and thrombotic thrombocytopenic purpura [2, 3].

Domestic ruminants, including cows, are the natural reservoirs of *E. coli* O157:H7, and they play a significant role in the epidemiology of human infections [4]. *E. coli* O157:H7 is carried in the intestinal tract, excreted in animal waste and may be transferred to carcasses during slaughter and evisceration. Human infection is via the faecal–oral route. This often results from ingestion of contaminated and improperly cooked meat and meat products [4].

Nigeria ranks first in the health burden of zoonotic diseases in Africa [5]. It was categorized into a sub-region that experiences the highest burden of foodborne disease globally, with *E. coli* O157 identified as one of the leading causes of foodborne disability adjusted life years [6].

The use of antibiotics for the treatment of *E. coli* O157:H7 infections has been very controversial. A study had reported that antibiotics may worsen the illness and increase the risk of haemolytic uremic syndrome, due to increased toxin production [7]. In contrast, early administration of some antibiotics is reported to be effective [8]. However, the frequent misuse of antibiotics as a growth-promoting supplement in animal feed creates a huge pressure for the selection of antibiotic-resistant strains among pathogenic bacteria, and contributes significantly to the emergence and spread of antibiotic resistance [9].

The lack of surveillance systems for *E. coli* O157:H7, poor and sub-standard hygienic conditions, unhygienic slaughter practices in abattoirs and the common traditional practices of raw meat consumption by certain tribes in Nigeria, constitute the major factors that predispose the local tribes to high risk of exposure and may possibly spread the pathogen to other Nigerians. In addition, there is paucity of information regarding the epidemiology of *E. coli* O157:H7 in Nigeria.

To bridge this knowledge gap, this study was designed to (1) assess the level of carcass contamination with *E. coli* O157:H7 in beef at a commercial slaughterhouse in Moro region of Kwara State, Nigeria; (2) test the susceptibility of the isolates to a panel of antibiotic agents; and (3) assess the hygienic practices at the abattoir during the slaughtering process.

## Methods

### Description of study area

This study was conducted in Kwara State. It covers an estimated 32 500 km^2^ in the North Central Geopolitical Zone of Nigeria. It is home to over 2 million people spread across 16 Local Government Areas, the majority of whom are pastoralists [10] . The target population for this study was cows (*Bos taurus*) raised by pastoralists in the Moro region of Kwara State, Nigeria (8.9407° N, 4.7821° E). The region has a central municipal abattoir. This abattoir renders slaughter services to the region and serves as the main destination for many market women and restaurant owners. This abattoir is among the five largest in Kwara State. It has a capacity to slaughter approximately 150 cows per week. There is high demand for meat throughout the year, and this increases during religious festivals. The number of staff in the abattoir also fluctuates to accommodate this demand. At the time of this study, the study abattoir has more than 25 employees.

### Study design

A cross-sectional study was conducted in one of the largest abattoirs in Kwara State to determine the prevalence and antibiotic susceptibility patterns of *E. coli* O157:H7 in cow meat, faeces and environmental samples. Sampling was carried out on 13 different occasions for a period of 4 months to achieve the desired sample size for a 95 % confidence level and a precision rate of 5 %. In order to minimize the chance of sampling multiple animals from the same herd, a systematic random sampling method was used to select animals at every visit. At each visit, the samples were selected at a pre-set interval, calculated by dividing the larger cow population by the desired sample size. A random starting point was chosen, and the sampling interval was repeated to choose subsequent samples. The sample size was determined by the formula of Thrusfield using a 5 % prevalence of *E. coli* O157:H7 in cow meats [11]. A total of 180 samples were collected for this study in order to maximize precision.

## Sample collection

### Carcass sampling

A total of 180 carcass swabs were collected. For each animal, samples were obtained from 4 different sites of the carcass (thorax, brisket, flank and crutch) following the guidelines of the International Organization for Standardization (ISO) [12]. Carcass swabs were obtained by using sterile cotton-tipped swabs (2×3 cm), pre-soaked in 10 ml of buffered peptone water (Oxoid, Hampshire, UK). The swabs were horizontally and vertically rubbed across the carcass surface. Upon the completion of the rubbing process, the shaft was removed by pressing it against the inner wall of the test tube, leaving only the cotton swab in the test tube. A different swab was used on each sampling site of the carcass, and swabs from the four different sites were pooled into a screw-capped test tube containing 10 ml of sterile buffered peptone water and immediately transported in cold boxes to the laboratory.

### Caecal sampling

A total of 180 caecal contents were collected. An aseptic incision was made with surgical blade in the caecum to obtain a representative sample of 10 g of the caecal content. The faecal material was aseptically compressed and the resultant liquid was decanted in a sterile universal bottle. The samples were labelled and transported in cold boxes to the laboratory.

### Environmental sampling and assessment of slaughter hygiene

A total of 55 environmental samples were collected, consisting of 16 water samples, 25 hand swabs and 14 butcher knife swabs. Environmental samples were collected following the methods described by Dulo *et al*. [13]. Both hands of abattoir workers were swabbed, including the blade and handle of the slaughter knives. In addition, 10 ml of water samples were collected before and during the slaughter process from the water buckets used for washing hands and cleaning equipment.

Slaughtering hygiene and the sanitary status of the abattoir were determined using a structured checklist, in addition to direct observations of the abattoir premises and the practices of the workers. The observational survey was conducted over the course of five visits. Five workers were observed at each visit for 15 min of work time, resulting in a total of 25 observed workers and 375 min of observational time.

Written permission to conduct the study was obtained from the management of the abattoir before the commencement of the study. A standard consent form was read to all potential study participants prior to their involvement in the study. Verbal consent was obtained because the majority of participants could neither read nor write. Only workers who gave verbal consent were included in the study. The verbal consent received from each study participant was indicated on the coded questionnaire by a square box. The management of the study abattoir served as witnesses. Overall, the study was approved by the Departmental Research Ethics Committee and the Centre for Community Development, Kwara State University (ref. no. BAB-02015-001).

Based on this survey results, our team organized and implemented a training package on hygienic practices for abattoir workers. The training was administered to workers of the study abattoir and results of the study were communicated during the training session.

### Culture and isolation of *E. coli* O157:H7

One millilitre of faecal pellet, carcass swabs and environmental samples were suspended into 9 ml of modified tryptone soya broth supplemented with novobiocin (10 mg l^−1^) (Oxoid Ltd, Hamsphire, UK). Samples were vortexed and incubated overnight at 37 °C. Aliquots of 100 µl from each enrichment broth were plated onto Sorbitol–MacConkey agar (Oxoid SR0172E) supplemented with 0.05 mg l^−1^ cefixime and 2.5 mg l^−1^ potassium tellurite (Oxoid) (CT-SMAC). The plates were incubated at 37 °C for 24 h. Multiple non-sorbitol fermenting colourless colonies from each plate were subcultured separately on CT-SMAC agar and incubated for 24 h at 37 °C for purification. After incubation, the purified and slightly transparent colourless colonies with a pale brownish appearance were tested for indole production as described by De Boer and Heuvelink [8]. Indole-forming colonies were then confirmed serologically by serotyping with antisera specific for the O157 and H7 antigens [12]. Serotyping was performed using the O157 and H7 Latex Test kit per the manufacturer’s instructions (DR0620, Oxoid Ltd, Hamspshire, UK). The latex kit consisted a test (0621) and a control latex (0622). The control latex had both the positive (DR0623) and negative control suspension (DR0624) containing inactivated *E. coli* O157 and O116 cells, respectively.

### Antibiotic susceptibility testing

All *E. coli* O157:H7 isolates were tested for antibiotic susceptibility to a panel of eight commercially available antibiotic-impregnated discs (Oxoid): nitrofurantoin (30 µg), ampicillin (5 µg), ceftazidime (30 µg), gentamicin (10 µg), ciprofloxacin (5 µg), augmentin (30 µg), ofloxacin (5 µg) and cefuroxime (30 µg), using the disc diffusion method [14] . The selection of these antibiotics was based on their frequency of use in ruminants and the standard antibiotic susceptibility testing recommendations of the Clinical and Laboratory Standards Institute [14].

Each bacterial colony isolated from pure culture was inoculated into 5 ml of tryptone soya broth (Oxoid Ltd, Hamsphire, UK) and incubated at 37 °C for 6 h. The turbidity of the culture broth was adjusted using sterile saline solution until the equivalent of 0.5 McFarland standard (~3×10^8^ c.f.u. ml^−1^) was obtained. Mueller–Hinton agar (Becton, Dickinson and Company, USA) plates were prepared according to the manufacturer’s instructions, and a sterile cotton swab was used to inoculate the test organism on the Mueller–Hinton agar plates. Antibiotic discs were carefully and aseptically placed at equal distance on the inoculated agar plates and the plates were incubated at 37 °C for 24 h. Following this, the antibiotic inhibition zone diameters were measured and the results were interpreted as susceptible, intermediate or resistant using the breakpoints of the CLSI. *E. coli* ATCC 25922 was used as a quality control strain.

### Statistical analysis

R statistical software [15] was used for data analysis. The prevalence of *E. coli* O157:H7 in carcass swab, caecal contents and environmental samples was determined by dividing the number of positive samples by the total number of samples examined. Significant association between *E. coli* O157 carriage and type of sample (carcass vs caecal content) was assessed using Fisher’s exact test. Differences were considered significant at *P*<0.05.

## Results

### Prevalence of *E. coli* O157:H7

Out of the total 415 different samples examined, 16 (3.9 %) were contaminated with *E. coli* O157:H7 (Table 1). *E. coli* O157:H7 was isolated from 10 (5.6 %) carcass swabs, 4 (2.2 %) caecal content samples and 2 (12.5 %) water samples. There was no statistical significant difference in the proportion of positive *E. coli* O157:H7 isolates between carcass swabs and caecal contents.

**Table 1.** Prevalence of *E. coli* O157:H7 at a commercial abattoir in Moro region of Kwara State, North-Central Nigeria

Sample types	No. of samples examined	No. of positive	% positive
Carcass swab	180	10	5.6
Caecal content	180	4	2.2
Water	16	2	12.5
Hand swab	25	0	0
Knife swab	14	0	0
**Total**	**415**	**16**	**3.9**

### Antibiotic susceptibility profile of *E. coli* O157:H7

Antibiotic susceptibility testing results showed that all the 16 isolates were resistant to ampicillin, ceftazidime and cefuroxime. Further, 14 (87.5 %) of the isolates were resistant to augmentin, 8 (50 %) were resistant to nitrofurantoin and 4 (25 %) were resistant to ciprofloxacin and gentamicin. None of the isolates were resistant to ofloxacin. All isolates were resistant to at least three antibiotics (Table 2).

**Table 2.** Antibiotic susceptibility patterns of *E. coli* O157:H7 isolates

Class and antibiotics	Concentration (μg)		no. of isolates, *n* (%)	
		Sensitive	Intermediate	Resistant
Nitrofurantoin	30	4 (25.0)	4 (25.0)	8 (50.0)
**Penicillin**				
Ampicillin	5	–	–	16 (100.0)
Augmentin	30	2 (12.5)	–	14 (87.5)
**Cephalosporin**				
Ceftazidime	30	–	–	16 (100.0)
Cefuroxime	30	–	–	16 (100.0)
**Aminoglycosides**				
Gentamicin	10	8 (50.0)	4 (25.0)	4 (25.0)
**Fluoroquinolones**				
Ciprofloxacin	5	11 (68.8)	1 (6.3)	4 (25.0)
Ofloxacin	5	16 (100.0)	–	–

### Facilities and practices at the abattoir

The observational surveys identified deficiencies in the abattoir facilities that hindered the maintenance of good hygiene (Table 3). The abattoir had no tap water, disinfectant, cooling facilities, toilet and bathroom facilities. All cows were slaughtered in the same area without any distinction between slaughter area and walk zones. Butchers were also observed using the same buckets of water to clean their knives, wash their hands, wash carcasses and even wash slaughter slabs.

**Table 3.** Results of observational survey on abattoir hygiene

Checklist	Sanitary hygiene conditions	Status
**Workers’ hygiene**	Use of protective clothing	0/25 (0 %)
	Slaughter knife is clean	0/25 (0 %)
	Clean water in buckets for washing	25/25 (100 %)
	Regular handwashing during work	0/25 (0 %)
	Received prior job-related training on hygiene	4/25 (16 %)
**Meat preservation**	Refrigerator in slaughter hall	Absent
	Separation of offal content from beef meat	19/25 (76 %)
**Slaughter hall hygiene**	Running water in slaughter hall	Absent
	Disinfectant in slaughter hall	Absent
	Use of hot water	2/25 (8 %)
	Separation of slaughter slabs and walk areas	Absent
	Soap in slaughter hall	Absent
**Toilet facilities**	Toilet and bathroom on premises	Absent

## Discussion

With reference to the literature search conducted prior to the execution of this project, this was actually the first study that investigated the prevalence and antibiotic susceptibility of *E. coli* O157:H7 in cows slaughtered at a major abattoir in the remote Moro region of Kwara State, North-Central Nigeria.

Comparatively, the prevalence of *E. coli* O157:H7 in carcass swabs (3.9 %) is consistent with the earlier findings of Dulo *et al*. [13] and Enabulele and Ndaka [16], which indicated a prevalence of 3.2 and 2.32 % in Ethiopian and Nigerian beef samples, respectively. However, Hika *et al*. [17] and Mershal *et al*. [18] obtained a higher prevalence in Central Ethiopia from meat samples (9.3 %) and carcass swabs (8.1 %), respectively. These variations in prevalence might be explained by methodological differences, such as the sampling points during the slaughtering process, the sampling season or the antibiotics administered, if any [19]. The intrinsic prevalence of *E. coli* O157:H7 is dependent on various factors, including the hygienic conditions of the farms and the surrounding environments [20]. *E. coli* O157:H7 could be present in water or grass consumed by the animals, particularly because Nigeria cattle farmers practice the free ranch system. Hence, the prevalence can vary extensively with different environmental sources.

The slaughter practices and hygienic conditions at the abattoir were conducive to cross-contamination of cow carcasses with *E. coli* O157:H7. Consequently, raw ground beef products present significant public health risks in Nigeria because of the common practice of some elites in consuming meat after they have only been cooked to an extra-rare or rare degree of doneness, which does not destroy the *E. coli* O157:H7 pathogen.

Further, the prevalence estimate for *E. coli* O157:H7 in cow carcasses (5.6 %) could potentially have resulted from cross-contamination with caecal content. Unhygienic practices during the slaughtering process, including the practice of some workers not aseptically separating the offal from beef, were observed in this study. Therefore, it is imperative to avoid carcass contamination during slaughter process in order to limit exposure to foodborne illnesses due to consumption of contaminated beef.

A prevalence estimate of 12.5 % was obtained from the abattoir wash water. The survey results further revealed that wash water was a major source of contamination and could facilitate carcass-to-carcass spread of pathogens across the slaughter line. Additionally, the observational survey revealed that the abattoir workers had not received adequate job-related training in meat handling and slaughter hygiene.

Personnel handling carcasses were not aware of the possibility of microbial cross-contamination from faecal material, intestines, their own hands, or the water used for cleaning. This was evident by their use of the same equipment for handling carcasses and even stripes. Cows were also slaughtered on the abattoir floor and butchered on slabs that were not adequately disinfected between carcasses. Butchers and retailers were also sighted walking between carcasses as they executed their business transactions. Further, the abattoir lacked tap water, toilet and bathroom facilities. Facilities for maintaining meats at safe temperatures were also absent. This is not surprising, as many of the public slaughterhouses in Nigeria are not well funded, with few or no resources allocated for the provision of necessary facilities that could enhance the maintenance of standard hygienic conditions.

It is paramount to note that the current level of antibiotic resistance in human pathogens is quite alarming and the rate at which this is now extending to pathogens and even commensals from animals is very worrisome [1, 21, 22]. In this study, multiple drug resistance was observed in all the isolates of *E. coli* O157:H7, with all the isolates exhibiting resistance to three or more antibiotics. Similar findings on multiple drug resistance of *E. coli* O157:H7 have been reported from other parts of the world [1, 23, 24]. The high prevalence of isolates (87.5–100 %) resistant to ampicillin, ceftazidine, cefuroxime and augmentin found in this study is in agreement with Tafida *et al*. [23], who reported resistance of *E. coli* O157:H7 isolates from retailed beef and related meat products to some of the above antibiotics, especially ampicillin.

One of the factors that has created a huge pressure for the selection of antibiotic resistance among pathogenic bacteria is the extensive and frequent misuse of antibiotics in agriculture and human and veterinary medicine. Many of these antibiotics are used as growth-promoting supplements in animals’ food and feed and this plays a significant role in the emergence and spread of antibiotic-resistant organisms [1] .

The significance of conducting antibiotic susceptibility profiling of clinical isolates and the need for regular surveillance for resistant pathogens cannot be over-emphasized, as these are crucial for the optimal therapy of bacterial-infected patients. The alarming rate at which multidrug-resistant organisms are emerging also requires more stringent monitoring and regulation of the use of antibiotics in animal and human populations

There are some limitations in this study. The genetic determinants and virulence factors for *E. coli* O157:H7 were not investigated due to limitations in laboratory facilities. Also, samples from the operators of the abattoir were not examined, and the possible sources of human contamination were not investigated. Further, quite a large number of mechanisms involving mobile genetic elements, such as plasmids, transposon and integrons, have been shown to contribute significantly to the spread of antibiotic resistance genes between bacterial organisms [21, 24]. However, due to the limitations in laboratory facilities, it was difficult to ascertain whether integrons or other mobile elements played a role in the multidrug resistance profiles observed in this study. This may form the basis for future research work on the *E. coli* O157:H7 isolates. Nonetheless, this study suggests that foodborne illness due to *E. coli* O157:H7 potentially constitutes a significant public health problem in the Moro region of Kwara State, Nigeria.

## Conclusion

The isolation of *E. coli* O157:H7 in meats slaughtered at the abattoir indicate the potential threat of the pathogen to public health. The study also revealed a high frequency of *E. coli* O157:H7 resistance to antibiotics commonly used in human and veterinary medicine. Monitoring resistance in enterohaemorrhagic *E. coli* O157:H7 is critical to stemming the risk of transmitting resistant strains from animals to humans, and the likelihood of horizontal transfer of resistance genes to other pathogens.

In line with the above conclusion, increasing awareness regarding meat safety, improving abattoir facilities and implementing hazard analysis critical control point (HACCP) practice are essential. Researchers should work with local health authorities to implement efficient epidemiological surveillance for prompt detection and control of *E. coli* O157:H7 in the community. Understanding the threat of *E. coli* O157:H7 to public health and food safety is critical to developing intervention strategies that can minimize the likelihood of an outbreak associated with meat consumption.
